# Toll-like receptor 4 is required for α-synuclein dependent activation of microglia and astroglia

**DOI:** 10.1002/glia.22437

**Published:** 2013-03

**Authors:** Lisa Fellner, Regina Irschick, Kathrin Schanda, Markus Reindl, Lars Klimaschewski, Werner Poewe, Gregor K Wenning, Nadia Stefanova

**Affiliations:** 1 Division of Neurobiology, Innsbruck Medical UniversityAnichstrasse 35, 6020 Innsbruck, Austria; 2 Department of Neurology, Innsbruck Medical UniversityAnichstrasse 35, 6020 Innsbruck, Austria; 3 Division of Neuroanatomy, Department of Anatomy, Histology and Embryology, Innsbruck Medical UniversityMuellerstrasse 59, 6020 Innsbruck, Austria

**Keywords:** alpha-synuclein, TLR4, oxidative stress, neuroinflammation

## Abstract

Alpha-synucleinopathies (ASP) are neurodegenerative disorders, characterized by accumulation of misfolded α-synuclein, selective neuronal loss, and extensive gliosis. It is accepted that microgliosis and astrogliosis contribute to the disease progression in ASP. Toll-like receptors (TLRs) are expressed on cells of the innate immune system, including glia, and TLR4 dysregulation may play a role in ASP pathogenesis. In this study we aimed to define the involvement of TLR4 in microglial and astroglial activation induced by different forms of α-synuclein (full length soluble, fibrillized, and C-terminally truncated). Purified primary wild type (TLR4^+/+^) and TLR4 deficient (TLR4^−/−^) murine microglial and astroglial cell cultures were treated with recombinant α-synuclein and phagocytic activity, NFκB nuclear translocation, cytokine release, and reactive oxygen species (ROS) production were measured. We show that TLR4 mediates α-synuclein-induced microglial phagocytic activity, pro-inflammatory cytokine release, and ROS production. TLR4^−/−^ astroglia present a suppressed pro-inflammatory response and decreased ROS production triggered by α-synuclein treatment. However, the uptake of α-synuclein by primary astroglia is not dependent on TLR4 expression. Our results indicate the C-terminally truncated form as the most potent inductor of TLR4-dependent glial activation. The current findings suggest that TLR4 plays a modulatory role on glial pro-inflammatory responses and ROS production triggered by α-synuclein. In contrast to microglia, the uptake of alpha-synuclein by astroglia is not dependent on TLR4. Our data provide novel insights into the mechanisms of α-synuclein-induced microglial and astroglial activation which may have an impact on understanding the pathogenesis of ASP. © 2012 Wiley Periodicals, Inc.

## INTRODUCTION

α-Synucleinopathies (ASP), including Parkinson's disease (PD), dementia with Lewy bodies (DLB), and multiple system atrophy (MSA), are characterized by α-synuclein (AS) positive cytoplasmic inclusions in neuronal and glial cells. AS is accepted to play a major role in the pathogenesis of ASP as suggested in different genetic (Al-Chalabi et al.,^2009^; Gasser^2009^; Scholz et al.,^2009^) and experimental studies (Shults et al.,^2005^; Stefanova et al.,^2001^,^2003^; Xilouri et al.,^2009^; Zhang et al.,^2005^). However, recent observations showed a crucial contribution of activated microglial and astroglial cells in ASP (Gerhard et al.,^2003^,^2006^; Hirsch et al.,^2005^; Ozawa et al.,^2004^).

Microglial cells are major effector cells of innate immunity in the central nervous system (CNS) (Streit,^2002^). They react to different stimuli such as injury, neurodegeneration, stroke, or brain tumors (Prinz et al.,^2011^; Stoll and Jander,^1999^; Streit,^2002^). Microglial cells remain in a quiescent state in the healthy brain and they survey the surrounding tissue for injury or invaders. In case of injury or infection, microglia can switch to an activated and phagocytic state, defined by morphological changes, proliferation, increased production of neurotrophic [e.g., brain-derived neurotrophic factor (BDNF)] or inflammatory factors [e.g., tumor necrosis factor-α (TNF-α)], and enhanced oxidative stress [e.g., reactive oxygen species (ROS) production] (Fellner et al.,^2011^; Nimmerjahn et al.,^2005^). Astroglia are the most numerous glial cell type in the CNS and display various important functions, such as support of synaptic transmission by control of extracellular homeostasis (Faissner et al.,^2010^; Fellner et al.,^2011^), maintenance of the blood–brain barrier (Simard and Nedergaard,^2004^), and regulation of the blood flow (Koehler et al.,^2009^). Furthermore, astroglia react to various CNS insults, such as infection, injury, and neurodegeneration, with morphological changes and variations in the molecular expression pattern (Wilhelmsson et al.,^2006^). In ASP, both cell types can develop over-activated phenotypes, which are termed microgliosis or astrogliosis and lead to chronic neuroinflammation (Fellner et al.,^2011^; Gerhard et al.,^2003^,^2006^; Hirsch et al.,^2005^; Ozawa et al.,^2004^).

Several studies explored AS-dependent activation of microglial cells (Austin et al.,^2006^; Klegeris et al.,^2008^; Reynolds et al.,^2008^; Rojanathammanee et al.,^2011^; Su et al.,^2008^). Especially mutant or aggregated forms of AS were shown to cause enhanced activation of microglial and astroglial cells, associated with cytokine release and oxidative stress (Klegeris et al.,^2006^; Lee et al.,2010a,b,c; Rojanathammanee et al.,^2011^; Roodveldt et al.,^2010^). Furthermore, recent evidence suggests that microglial and astroglial reactivity contribute to dopaminergic degeneration (Rappold and Tieu,^2010^; Saijo et al.,^2009^; Zhang et al.,^2005^), and may mediate the progression of neurodegeneration in ASP (Fellner et al.,^2011^; Halliday and Stevens^2011^).

Although there is evidence to suggest a significant contribution of microgliosis and astrogliosis in the pathogenesis of ASP, the mechanisms underlying microglial and astroglial activation in these diseases are largely unknown. An involvement of toll-like receptors (TLRs) in ASP is presumed, based on the recently found up-regulation of TLRs in ASP (Letiembre et al.,^2009^; Stefanova et al.,^2007^). TLRs belong to the family of pattern recognition receptors and are crucial players in the innate immune response. They recognize pathogen-associated molecular patterns [e.g., lipopolysaccharide, LPS] and endogenous molecules, including misfolded proteins (Akira^2001^; Glezer et al.,^2007^; Lehnardt^2010^). TLRs are expressed on innate immune system cells, including microglial and astroglial cells (Akira^2001^; Alfonso-Loeches et al.,^2010^; Bowman et al.,^2003^; El-Hage et al.,^2011^; Kielian^2006^). TLR4 signaling leads to translocation of nuclear factor (NF)-κB to the nucleus and expression of pro-inflammatory cytokines (Okun et al.,^2009^). Recent findings suggest that TLR4 may be involved in the pathogenesis of ASP. Up-regulation of TLR4 was shown in ASP postmortem tissue as well as in a transgenic mouse model (Letiembre et al.,^2009^; Stefanova et al.,^2007^). Experimental TLR4 deficiency led to decreased AS clearance by murine microglia (Stefanova et al.,^2011^). However, the role of TLR4 in AS-dependent activation of microglial and astroglial cells remains unclear.The main goal of this study was to determine the role of TLR4 in AS-dependent activation of microglial and astroglial cells. Therefore, we conducted cell culture assays to analyze the effect of three different AS forms (full length soluble, fibrillized, and C-terminally truncated) on wild type (TLR4^+/+^) and TLR4 deficient (TLR4^−/−^) microglia and astroglia. Exposure of microglia with TLR4 ablation to AS resulted in reduced phagocytic activity, decreased ROS production, and diminished release of pro-inflammatory cytokines. Similarly the pro-inflammatory astroglial response was suppressed by TLR4 deficiency, however the uptake of AS by astroglia was not affected by TLR4 ablation.

## MATERIALS AND METHODS

### Preparation, Purification, and Characterization of Full Length Soluble, Fibrillar, and C-Terminally Truncated AS Proteins

The human full length AS (aa 1-140) was amplified from human spinal cord cDNA (Clontech, Palo Alto, CA) using polymerase chain reaction (PCR) as previously described (Stefanova et al.,^2001^). We used the following primers for full length AS: sense primer 5′-CAC CAT GGA TGT ATT CAT GAA AG-3′, antisense primer 5′-GGC TTC AGG TTC GTA GTC TTG-3′; and for C-terminally truncated AS (aa 1-111): sense primer 5′-CAC CAT GGA TGT ATT CAT GAA AG-3′, antisense primer 5′-TCC TTC CTG TGG GGC TC-3′ (Microsynth, Balgach, Switzerland). Cloning, sequence verification, protein expression, purification by affinity chromatography, dialysis, and endotoxin removal of the probes were accomplished as described before (Stefanova et al.,^2011^). The protein preparations were filter sterilized through a 0.2-μm filter, and stored at −80°C. Protein content was measured using the BCA protein assay (Sigma-Aldrich, St. Louis, MO). For further experiments, formation of oligomeres and fibrils was induced by incubation of full length AS at 37°C for 2 weeks (fAS) (Zhang et al.,^2005^). It is known that AS is able to self-assemble under certain conditions (Cole et al.,^2002^), including incubation at 37°C (Conway et al.,^2000^). All AS forms were verified by immunoblotting, using NuPAGE 10% Bis-Tris gels (Invitrogen, Carlsbad, CA) for protein separation. Proteins were electrotransferred to a nitrocellulose membrane (GE Healthcare Bio-Sciences AB, Uppsala, Sweden) and after blocking with 2% milk powder in PBS containing 0.05% Tween-20 (PBS-T), the blots were incubated with the purified monoclonal AS antibody (aa 15-123, 1:1000, BD Transduction Laboratories, San Jose, CA). Blots were further incubated with alkaline phosphatase linked anti-mouse IgG (1:5000, Jackson Immunoresearch Laboratories, West Grove, PA) and developed using NBT/BCIP (Roche, Vienna, Austria). The fibrillization of AS was verified by Thioflavin T (ThT) fluorescence as described previously (Apetri et al.,^2006^; Bolder et al.,^2007^). Briefly, full length AS was incubated for up to 20 days at 37°C. 10 μL samples were added to 1 mL 20 μM ThT (Sigma-Aldrich) in PBS and mixed well. Fluorescence emission spectra were immediately recorded at 465–600 nm with excitation at 440 nm as described elsewhere (Apetri et al.,^2006^). Control measurements were performed with 20 μM ThT in PBS and background fluorescence intensity (*I*_0_) was defined. The fluorescence intensity of each sample (*I*_ThT_) was normalized to *I*_0_ according to the formula *I*_ThT_* = (*I*_*ThT*_ − *I*_0_)/*I*_0_) as previously described (Bolder et al.,^2007^) and finally plotted versus the time of incubation at 37°C.

Finally, to exclude potential contamination the endotoxin concentration in the different AS preparations (full length soluble, fibrillized, and C-terminally truncated) was determined by Hyglos GmbH, Bernried, Germany using the kinetic chromogenic Limulus Amoebocyte Lysate (LAL) endpoint assay. The amount of endotoxin in the stock solutions was under 1 EU/mg, i.e., a concentration that is incapable of inducing significant glial activation as previously reported (Gao et al.,^2003^; Lee et al.,2010a; Park et al.,^2008^; Zhang et al.,^2005^).

### Mouse Primary Microglial and Astroglial Cultures

Mouse purified primary microglial and astroglial cultures were prepared from brains of wild type (TLR4^+/+^, C57BL/6) and TLR4 deficient (TLR4^−/−^, C57BL/10ScNJ, Jackson Laboratories, Sacramento, CA, stock No. 003752) newborn mouse brains (Days 1–3) as described previously (Stefanova et al.,^2011^). Briefly, mice were sacrificed and brains were isolated and cortices prepared. Meninges were removed, cortices minced and cells were dissociated. Cells were suspended in Dulbecco's modified Eagle's medium: Nutrient mixture F-12 (DMEM/F12, Gibco, Invitrogen, Carlsbad, CA) including 2 mM L-glutamine, 10% fetal calf serum (FCS, Gibco), 100 U/mL penicillin, and 100 μg/mL streptomycin (Gibco), plated on precoated poly-D lysine (PDL, 20 μg/mL, Gibco) T75 flasks (TPP, Trasadingen, Switzerland) and incubated at 37°C in a humid atmosphere with 5% CO_2_. Purified microglial cells were gained by shaking the mixed glial cultures on an orbital shaker at 180 rpm overnight at 37°C in a humid atmosphere with 5% CO_2_. The purity of the microglial cells in the supernatant was determined by CD11b immunocytochemistry and by flow cytometry analysis (93% CD11b positive) as characterized previously (Stefanova et al.,^2011^). Cells were plated in DMEM (Gibco) including 20% FCS and 2 mM L-glutamine at a density of 100,000 cells/ well in 24-well cell culture plates (TPP) or 15,000 cells/well in 96-well cell culture plates (TPP) (Stefanova et al.,^2011^). Pure microglial cultures were exposed to AS or lipopolysaccharide (LPS, Sigma-Aldrich) 24 h post shaking.

For the generation of an aged astroglial culture, the mixed glial cultures were shaken overnight once a week. After three shaking cycles, the supernatant containing microglia was removed and the remaining astroglial layer was washed to further remove dead or loose cells. Astroglia were detached using Trypsin-EDTA (Gibco) and plated in 24-well cell culture plates at a density of 40,000 cells/ well or 20,000 cells/well in 96-well cell culture plates. After a week in culture, the confluent cell layer was shaken again for 1 h, followed by the replacement of the medium and another shake over night. Again the microglia containing medium was replaced with fresh medium and after 24 h in culture, experiments were started. The purity of the astroglial cell cultures (82%) was determined by GFAP immunocytochemistry. Furthermore, astroglial TLR4 expression was confirmed by TLR4 and GFAP immunocytochemistry (Supporting Information).

### Phagocytosis Assay

Phagocytic activity measurement of AS-stimulated microglia was performed as described elsewhere (Reed-Geaghan et al.,^2009^). Primary microglial cultures were challenged with 3 μM of full length soluble, fibrillar or C-terminally truncated AS (sAS, fAS, or tAS). After 24 h, phagocytic activity was determined using fluorescent microspheres (1 μm, Invitrogen) and compared with untreated controls. After incubation, cells were fixed with 4% paraformaldehyde (PFA, Merck, Vienna, Austria), over 150 cells per treatment were analyzed and the percentage of phagocytic cells was determined. All measurements were repeated in five separate biological replicates. Values were averaged for all five experiments (±S.D.) for statistical analysis.

### Uptake of Recombinant AS

To investigate the uptake of recombinant AS in relation to TLR4 expression, primary astroglial cells (TLR4^+/+,^ and TLR4^−/−^) were plated onto PDL-coated 4-well cover slips (Thermo Scientific) or 24-well plates. The cells were treated 2 h with 3 μM recombinant AS as described previously (Stefanova et al.,^2011^). Cells were fixed with 4% PFA, immunostained against AS and GFAP, and AS uptake was determined.

### Indirect Immunofluorescence Staining

The following primary antibodies were used in this study: monoclonal rat anti-mouse CD11b (1:100, Serotec, Oxford, UK), monoclonal mouse anti-glial fibrillary acidic protein (1:500, GFAP, Millipore, Temecula, CA), LEAF™ purified rat anti-mouse Toll-like Receptor 4 (TLR4, CD284)/MD2 Complex (1:50, BioLegend, San Diego, CA), rat anti-human AS (aa 116-131 hAS, 1:500, 15G7, Enzo Life Sciences, Loerrach, Germany), mouse anti-AS (aa 15-123, 1:100, BD Transduction Laboratories, San Jose, CA) and rabbit anti-mouse NF-κB (1:200, Abcam, Cambridge, UK). After washing, cells were fixed with 4% PFA followed by 1 h blocking with solution containing 0.3% Triton-X100, 1% bovine serum albumin, and 5% normal serum (from goat or horse as appropriate) in PBS. Cells were then incubated with primary antibody overnight at 4°C, and secondary antibody for immunofluorescence, including Alexa 488- or Alexa-594-conjugated anti-rat, anti-rabbit, or anti-mouse IgG (Jackson Immunoresearch Laboratories, West Grove, PA) for 1 h at room temperature (RT). Accordingly, negative controls by omitting the primary antibody and using only the secondary antibodies were performed for each experiment. Nuclear staining of fixed cells was performed with 4',6-diamidino-2-phenylindole dihydrochloride (DAPI, Sigma-Aldrich). DAPI was diluted 1:20,000 and cells were incubated for 3–5 min at RT. Cells were visualized using a DMI 4000B Leica inverse microscope and Application Suite V3.1 and Digital Fire Wire Color Camera DFC300 FX by Leica or using confocal microscopy.

### Confocal Microscopy

Confocal microscopy was performed with Leica TCS SP5 laser scanning microscope (Leica Microsystems, Wetzlar, Germany) with a 63x glycerol objective (N.A. 1.3) and a pinhole of 1 AU. Z-stacks for 3D-reconstruction of images were acquired according to the Nyquist criterion. Image deconvolution was performed with Huygens Professional software version 4.1.1 (SVI Scientific Volume Imaging, Hilversum, NL). Images were processed with the Huygens Object Analyzer Advanced and cells were reconstructed three-dimensionally with the Huygens MIP Renderer.

### Measurement of ROS

Measurement of intracellular superoxide radical generation by the formation of a dark blue formazan deposit resulting from superoxide-mediated reduction of NBT (nitroblue tetrazolium chloride, Roche Applied Sciences) was approached as previously described (Reed-Geaghan et al.,^2009^). Briefly, primary TLR4^+/+^ and TLR4^−/−^ glial cells (microglia, astroglia) were plated in 24-well-plates and challenged with 2 μM of different AS forms (sAS, fAS, and tAS) for up to 48 h. Untreated cells were used as controls. One mg/ml NBT was added at 37°C for 30 minutes. Cells were fixed with 4% PFA at RT. Over 500 cells per treatment were analyzed and the percentage of ROS-positive cells was determined using a DMI 4000B Leica inverse microscope. All measurements were repeated in three separate biological replicates and mean values (±S.D.) were determined.

### Measurement of Cytokine Release by Fluorometric Multiplex Bead-Based Immunoassay

FlowCytomix measurements using the mouse TNF-α, CXCL1, IL-6, IL-1α, IL-1β, and GM-CSF simplex kits and the mouse basic kit (all by BenderSystems, Vienna, Austria) were performed according to the manufacturer's protocol in supernatants from purified microglial and astroglial cell cultures treated with 3 μM of different AS forms (C-terminally truncated, fibrillar, soluble) over 2, 12, and 24 h. The supernatants of untreated and LPS (100 pg/mL) treated microglia and astroglia were used as controls. All measurements were repeated in three separate biological replicates and the mean values (±S.D.) were determined for statistical analysis.

### Statistical Analysis

All statistical analyses were carried out using GraphPad Prism 5 (Graphpad Software, San Diego, CA) and the results were presented as the mean ± S.D. One-way analysis of variance for multiple comparisons, and two-way analysis of variance with post-hoc Bonferroni test for the analysis of two independent factors (e.g., genotype and treatment), were applied. A *P* value <0.05 was considered statistically significant.

## RESULTS

### TLR4 Is Required for AS-Induced Phagocytic Response in Primary Microglia

**Figure 1:**
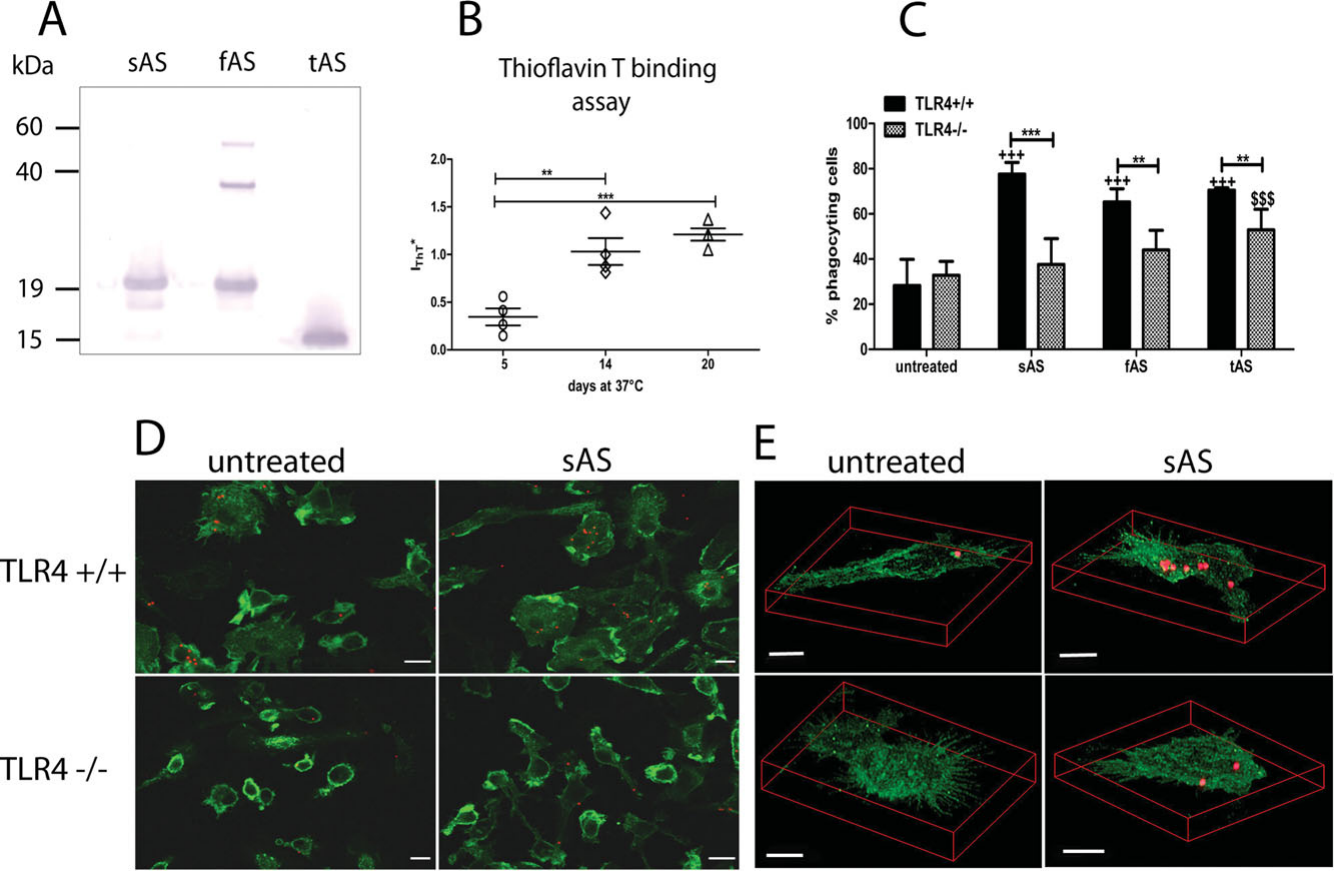
Microglial phagocytic activity is increased after AS-treatments, but diminished in TLR4-deficient microglia. Initially, the purity and oligomeric state of the recombinant AS preparations was determined. A: Immunoblot was performed of full length soluble, fibrillized (14-day old), and C-terminally truncated AS (sAS, fAS, and tAS). In addition to the monomer band fAS shows different oligomer species. B: The fibrillization of AS incubated at 37°C was controlled using the Thioflavin T assay. The amount of fibrillized AS was significantly augmented after 14 days at 37°C. No significant change in the degree of fibrillization was detected between AS probes after 14-day and 20-day incubation. Therefore, 14-day-old fibrillized AS (fAS) was used for all the experiments. Data were analyzed by one-way analysis of variance for multiple comparisons. ***P < 0.001, **P < 0.01 compared with 5-day incubation. Next, primary murine microglia were treated with 3 μM sAS (full length soluble AS), fAS (full length fibrillized AS) or tAS (C-terminally truncated AS), and fluorescent microspheres (red) were added. Cells were fixed with 4% paraformaldehyde and immunostained for CD11b (green). C: Morphometric analysis of CD11b-labeled microglia with incorporated microspheres revealed that AS treatment (all forms) led to a significant increase of phagocyting cells. TLR4 deficiency induced a decline of microglial phagocytic activity. Data were analyzed by two-way ANOVA with post-hoc Bonferroni test. Results are presented as mean ± S.D. +++*P* < 0.001 compared with untreated TLR4^+/+^ microglia, ***P < 0.001, **P < 0.01 comparison of TLR4^+/+^ to TLR4^−/−^ microglia, $$$*P* < 0.001 compared with untreated TLR4^−/−^ microglia. *n* = 5. D: Confocal microscopy of microglia revealed AS-dependent increase of phagocytosed microspheres by TLR4^+/+^ microglia (scale bar, 10 μm), whereas TLR4 ablated microglia featured reduced phagocytosis of fluorescent microspheres. Representative for all three treatments we show microglia treated with sAS and fluorescent microspheres. E: A representative confocal image stack confirmed the uptake of the microspheres (red) into CD11b-positive microglia, and further demonstrated that the amount of incorporated microspheres is decreased in TLR4^−/−^ microglia compared with TLR4^+/+^ microglia (scale bar, 5 μm).

The purified recombinant AS proteins were primarily characterized by immunoblotting. As expected full length soluble AS presented with a single band corresponding to the monomer size of 19 kDa. Full-length fibrillized AS showed additional immunoreactive products, corresponding to different oligomer species of about 40 and 60 kDa. The size of the C-terminally truncated AS form was 15 kDa (Fig. .1A). The fibrillization of AS was further verified using the ThT binding assay. After 14 days at 37°C, the amount of fibrillized AS was significantly elevated. After 20 days incubation at 37°C, we did not observe a significant increase of fibrils compared with the 14-day time point (Fig. .1B). Therefore we used the 14-day-old fibrillized AS in all further experiments.

Next, we evaluated microglial phagocytic activity upon AS treatment. We previously described loss of AS uptake in BV2 microglial cells after functional block of TLR4 as well as in TLR4 deficient primary microglia (Stefanova et al.,^2011^). Here we further investigated the effects of different extracellular AS forms and TLR4 deficiency on microglial phagocytic activity. We used an *in vitro* phagocytosis assay assessing the uptake of fluorescent microspheres by microglia (Reed-Geaghan et al.,^2009^). TLR4^+/+^ microglia showed augmented phagocytic activity after treatment with sAS, fAS, and tAS. Microglial phagocytic activity was significantly reduced in TLR4^−/−^ microglia at baseline and after treatment with different AS forms (Fig. .1C–E).

### AS Treatment Is Associated with NF-κB Nuclear Translocation in TLR4^+/+^, But Not in TLR4^−/−^ Microglia

**Figure 2:**
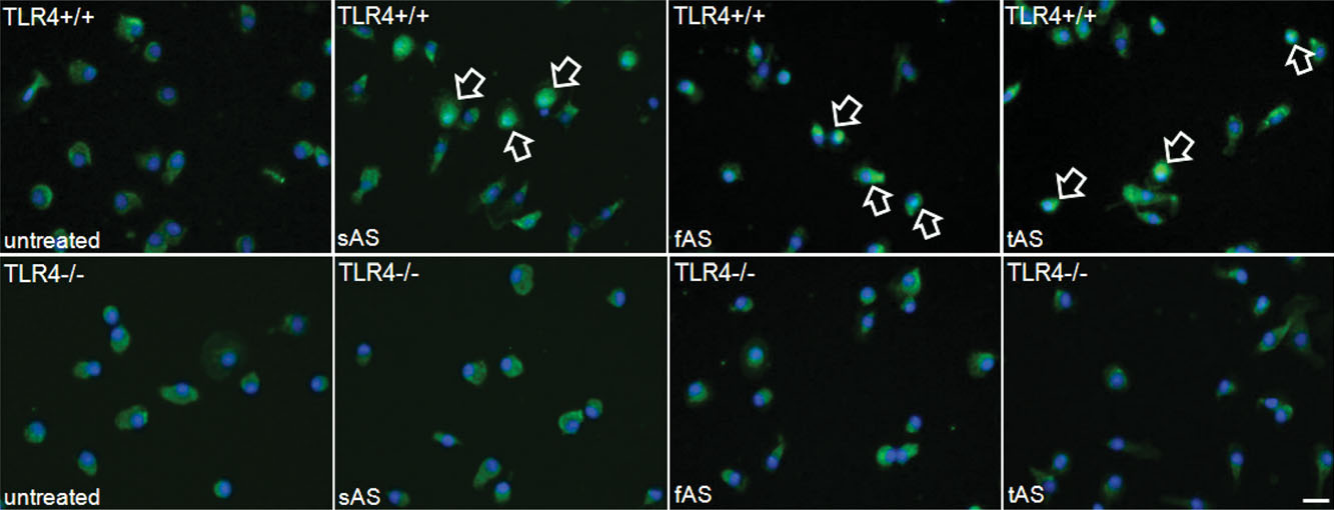
NF-κB nuclear translocation upon AS treatment in TLR4^+/+^ microglia. Murine primary microglia were treated with different AS-forms (full length soluble AS–sAS, fibrillized AS–fAS, and C-terminally truncated AS–tAS). Cells were fixed with 4% paraformaldehyde, immunostained for NF-κB (green) and counterstained with DAPI (blue). NF-κB translocation in TLR4^+/+^ microglia treated with AS was identified by co-localization of DAPI and NF-κB (see arrows). No co-localization was observed in untreated and AS treated TLR4^−/−^ microglia (scale bar, 10 μm).

TLR4 activation classically results in downstream activation of the transcription factor NF-κB. To identify NF-κB translocation into the nucleus 2 h after treatment with different forms of AS, we performed NF-κB immunofluorescence labeling of treated and untreated microglial cells, which were then counterstained with DAPI. We observed a clear co-localization of DAPI and NF-κB immunofluorescence indicating NF-κB translocation into the nucleus of AS treated TLR4^+/+^ microglia. However, no NF-κB nuclear translocation occurred in TLR4^−/−^ microglial cells after exposure to sAS, fAS, or tAS (Fig. .2). Since TLR4 signaling leading to nuclear translocation of NF-κB results in the induction of pro-inflammatory cytokines (Okun et al.,^2009^), we next addressed the inflammatory profile of TLR4^+/+^ and TLR4^−/−^ microglia upon AS exposure.

### TLR4 Ablation in Microglia Prevents Release of TNF-α, IL-6, and CXCL1 upon AS Treatment

**Figure 3:**
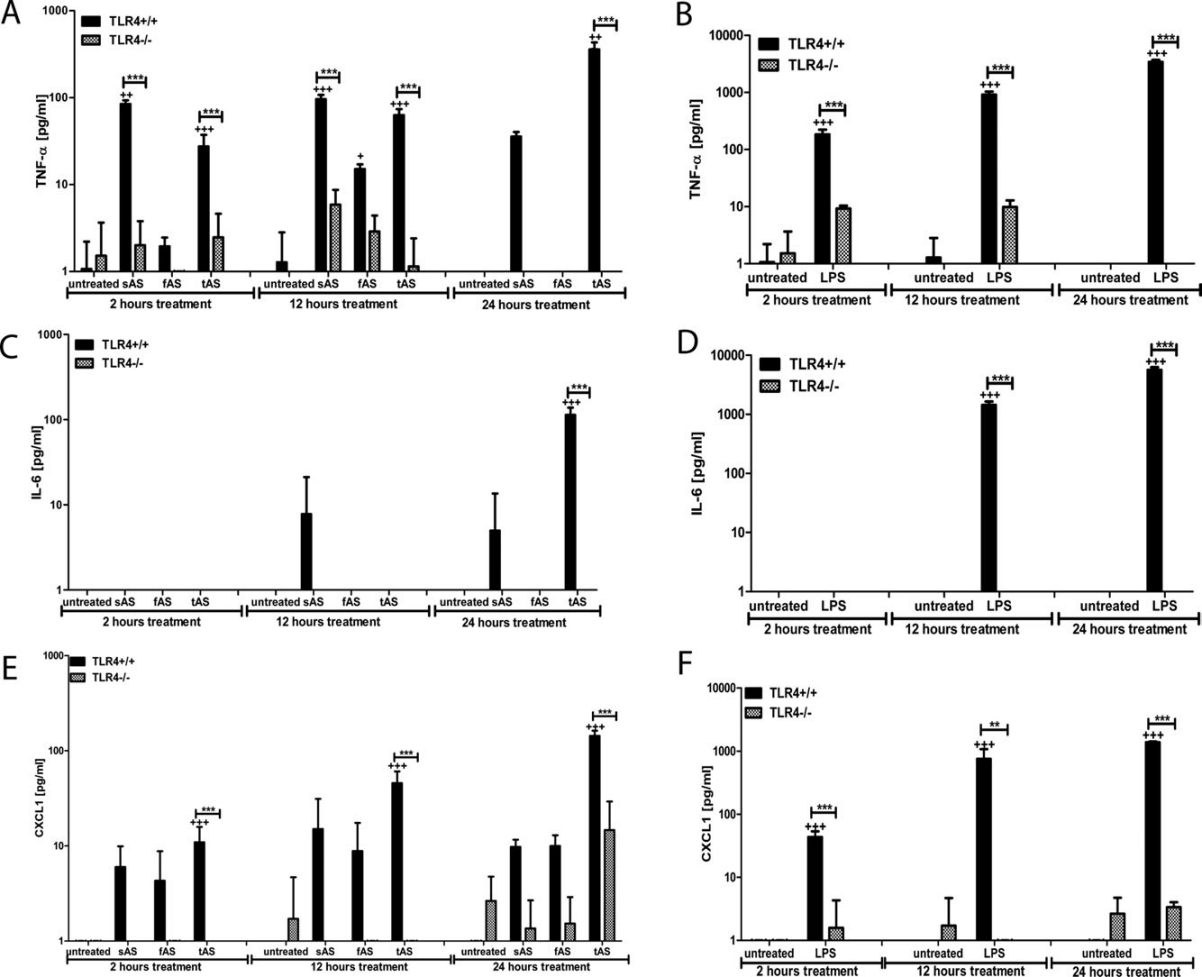
Inflammatory responses of TLR4^+/+^ and TLR4^−/−^ microglia upon AS treatment. Cytokine measurements were performed using the fluorometric multiplex bead-based immunoassay (BenderSystems). Primary murine microglia (TLR4^+/+^ and TLR4^−/−^) were treated with different AS forms (full length soluble AS–sAS, fibrillized AS–fAS, and C-terminally truncated AS–tAS) or LPS as positive assay control for 2, 12, and 24 h. The supernatants were collected and used for quantifying the amount of secreted TNF-α (A, B), IL-6 (C, D) and CXCL1 (E, F). TLR4 deficiency lead to a significantly decreased TNF-α, IL-6, and CXCL1 production, especially after tAS and control LPS treatment, whereas the treatment with tAS induced a significantly elevated TNF-α, IL-6, and CXCL1 production in TLR4^+/+^ microglia. Data were analyzed by two-way ANOVA with post-hoc Bonferroni test. Data are the average (±SD) of three independent experiments. +++ *P* < 0.001, + *P* < 0.05 compared with untreated TLR4^+/+^ microglia, ***P < 0.001 comparison of TLR4^+/+^ to TLR4^−/−^ microglia.

To establish a profile of microglial inflammatory response to extracellular sAS, fAS, and tAS, we analyzed the release of several pro-inflammatory cytokines in the supernatant at 2, 12, and 24 h of AS treatment. Out of all the tested cytokines in our experiments, the pro-inflammatory response of microglia to extracellular AS was characterized by release of TNF- α, IL-6, and CXCL1. We observed a significant early TNF-α release (after 2 h) by microglia upon treatment with sAS and tAS treatment (Fig. .3A). TNF-α production upon tAS treatment increased further up to 24 h, while TNF-α levels upon sAS treatment remained constant. Treatment with fAS-induced transient TNF-α release detected 12-h after exposure to fAS. TLR4 ablated microglia showed no or significantly reduced TNF-α production up to 24 h after treatment with the different AS forms (Fig. .3A). In comparison, control treatment with LPS led to a continuous increase of TNF-α production in TLR4^+/+^ microglia, but no TNF-α release by TLR4^−/−^ microglia exposed to LPS was detected (Fig. .3B).

IL-6 release by TLR4^+/+^ microglia was detectable only after a 24-h-treatment with tAS (Fig. .3C). In comparison, control LPS treatment of TLR4^+/+^ microglia caused IL-6 release after 12-h exposure, and IL-6 levels significantly increased with time (Fig. .3D). Similar to the TNF-α production profile, IL-6 release was not detectable in TLR4 ablated microglia neither at baseline, norunder exposure to sAS, fAS, tAS or control LPS (Fig. .3C,D).

Finally tAS treatment of TLR4^+/+^ microglia induced significantly elevated CXCL1 production which constantly increased over time. However, AS-treated TLR4 deficient microglia showed reduced or not detectable CXCL1 release at all studied time-points as compared with TLR4^+/+^ microglia (Fig. .3E). Control exposure to LPS induced enhanced and continuous CXCL1 release by TLR4^+/+^ microglia. This response to LPS was abolished in TLR4^−/−^ microglia (Fig. .3F).

### Reduced ROS Production in TLR4 Deficient Microglia and Astroglia upon AS Treatment

**Figure 4:**
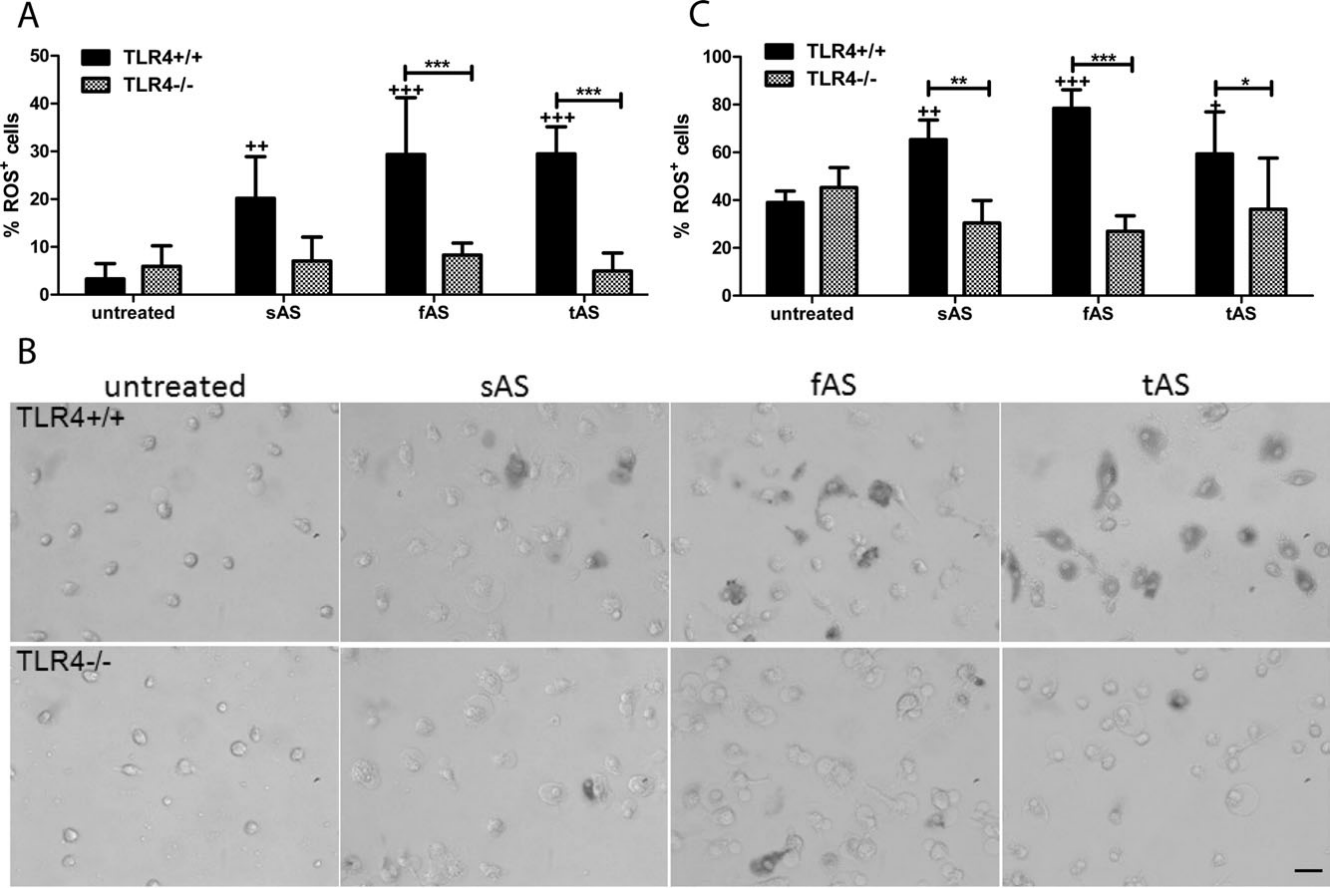
TLR4 ablation results in a reduction of ROS production by glial cells after exposure to AS. Primary murine microglial and astroglial cells were treated with different AS forms (full length soluble AS–sAS, fibrillized AS–fAS, and C-terminally truncated AS–tAS). Nitroblue tetrazolium chloride was added to visualize ROS production. Cells were fixed with 4% paraformaldehyde and ROS-positive cells were counted using an inverse microscope. A: Treatment with all AS-forms led to an enhanced ROS release by TLR4^+/+^ microglia. TLR4 deficient microglia showed a reduced production of ROS in comparison to TLR4^+/+^ microglia. Data were analyzed by two-way ANOVA with post-hoc Bonferroni test. Results are presented as mean ± S.D. +++ *P* < 0.001, ++ *P* < 0.01 compared with untreated TLR4^+/+^ microglia, ***P < 0.001 comparison of TLR4^+/+^ to TLR4^−/−^ microglia. *n* = 3 (excl. untreated TLR4^+/+^ and TLR4^−/−^ microglia *n* = 5). B: Light microscopy of microglia visualizing the difference of ROS production by TLR4^+/+^ and TLR4^−/−^ microglia (scale bar, 10 μm). C: Treatment with all AS forms initiated augmented ROS production by TLR4^+/+^ astroglia, whereas, TLR4 ablated astroglia showed a decreased ROS production in comparison to TLR4^+/+^ astroglia. Data were analyzed by two-way ANOVA with post-hoc Bonferroni test. Results are presented as mean ± S.D. +++ *P* < 0.001, ++ *P* < 0.01, + *P* < 0.05 compared with untreated TLR4^+/+^ astroglia, ***P < 0.001, **P < 0.01, *P < 0.05 comparison of TLR4^+/+^ to TLR4^−/−^ microglia *n* = 3.

AS was shown to promote oxidative stress (Fellner et al.,^2011^; Lee et al.,2010a; Zhang et al.,^2005^); therefore, we sought to identify the production of ROS by microglial and astroglial cells after AS treatment in our cell culture model. ROS positive cells were easily recognized by the visible blue precipitate in the cytoplasm resulting from superoxide-mediated reduction of NBT. We observed a significant up-regulation of ROS production in TLR4^+/+^ microglia (Fig. .4A,B) and astroglia (Fig. .4C) treated with sAS, fAS, and tAS as compared with untreated glial cells. A significantly decreased number of ROS positive cells was determined in TLR4 deficient microglia treated with tAS and fAS as compared with TLR4^+/+^ microglia upon the same treatment (Fig. .4A,B). Astroglial TLR4 ablation lead to a significant reduction of ROS positive astroglial cells after treatment with all AS forms (Fig. .4C).

### Uptake of Recombinant AS by Astroglial Cells is Not Dependent on TLR4

**Figure 5:**
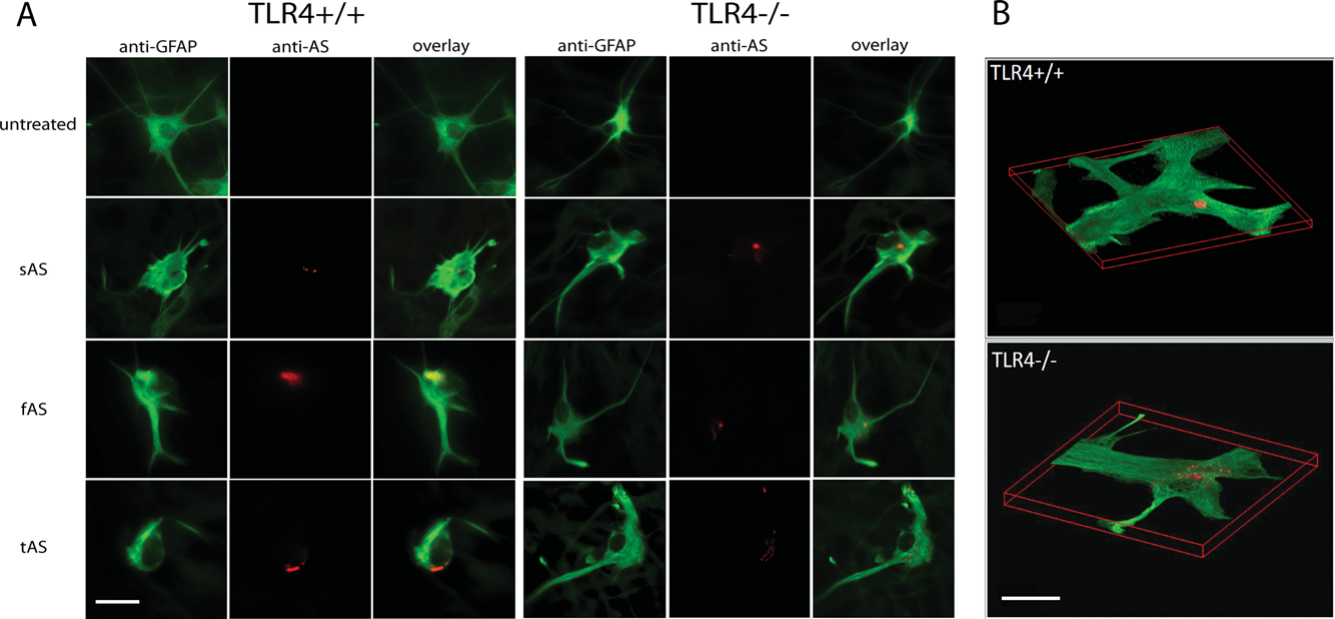
Uptake of AS by aged primary murine astroglial cells. Astroglial cells were treated with different AS-forms (full length soluble AS–sAS, fibrillized AS–fAS, and C-terminally truncated AS–tAS). Cells were fixed with 4% paraformaldehyde and immunostained for GFAP (green) and AS (red). A: Both TLR4^+/+^ and TLR4^−/−^ astroglial cells presented incorporated AS in the cytoplasm (scale bar 20 μm). B: Representative three-dimensionally reconstructed confocal images of GFAP-positive astroglia with incorporated sAS are shown (scale bar 10 μm).

Astroglial AS-positive inclusions are common in PD (Wakabayashi et al.,^2000^). Recent evidence suggests that astroglia endocytose neuronal cell-derived AS, causing an inflammatory response (Lee et al.,2010c). To evaluate if extracellular recombinant AS may be uptaken by aged primary murine astroglia *in vitro*, we exposed purified primary astroglia to different recombinant AS forms and identified AS uptake by immunocytochemistry and confocal microscopy. Two hours after treatment, AS-positive intracytoplasmic profiles were identified in both TLR4^+/+^ and TLR4^−/−^ GFAP-positive cells (Fig. .5), indicating that AS uptake in astroglia was not TLR4 dependent.

### tAS Most Potently Triggers TLR4-Dependent Release of TNF-α, IL-6, and CXCL1 by Astroglia

**Figure 6:**
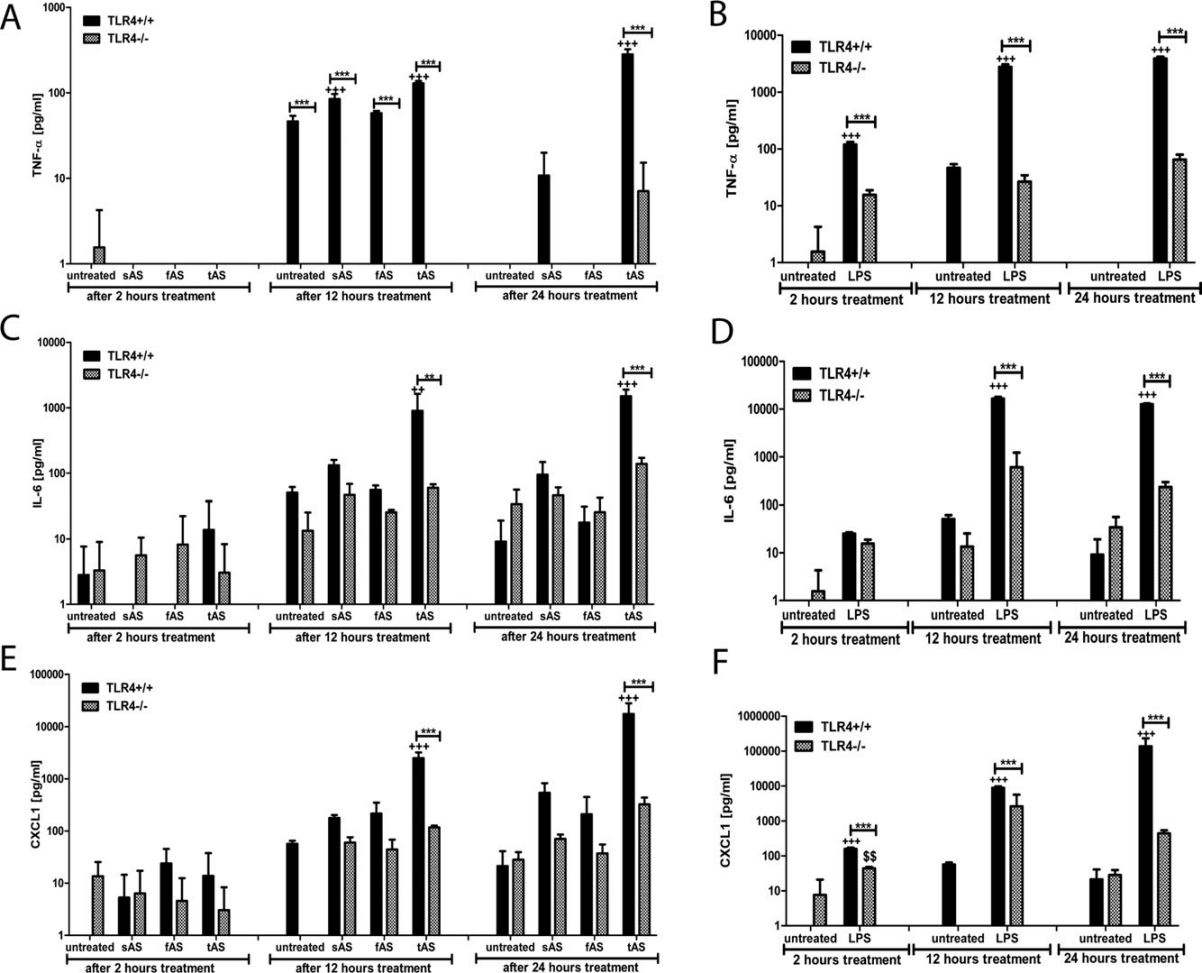
Analysis of cytokine and chemokine release upon AS treatment of aged primary astroglia. FlowCytomix analyses of TNF-α (A, B), IL-6 (C, D), and CXCL1 (E, F) were performed using supernatants of AS- or LPS-treated primary murine astroglial cells after 2, 12, and 24 h. Especially tAS caused a significant cytokine/chemokine release in TLR4^+/+^ astroglia. In comparison, TLR4 ablation reduced the released cytokine amount significantly. Data were analyzed by two-way ANOVA with post-hoc Bonferroni test. Results are presented as mean ± S.D. +++ *P* < 0.001 compared with untreated TLR4^+/+^ astroglia, *** *P* < 0.001, ** *P* < 0.01 comparison of TLR4^+/+^ to TLR4^−/−^ astroglia, and $$ *P* < 0.01 compared with untreated TLR4^−/−^ astroglia. *n* = 3.

It was previously postulated that AS uptake by astroglia is directly linked to the astroglial pro-inflammatory response associated with drastic increase in CXCL1 and TNF-α production (Lee et al.,2010c). For all the tested cytokines and chemokines in our experiments, detectable changes were found in CXCL1, IL-6, and TNF-α release by astroglia upon AS treatment. Significant TNF-α production by TLR4^+/+^ astroglial cells was detected 12 h after sAS and tAS treatment. TNF-α release further doubled after 24-h exposure to tAS, while diminishing after 24 h treatment with sAS (Fig. .6A). The release of IL-6 and CXCL1 was significantly augmented upon tAS treatment after 12 and 24 h (Fig. .6C,E). TLR4 ablation in astroglia abolished TNF-α release triggered by AS treatments and significantly reduced, IL-6 and CXCL1 release after tAS treatment. (Fig. .6A,C,E). As a positive control of the assay, astroglia were treated in parallel with LPS. LPS induced TNF-α and CXCL1 release already 2 h after treatment and was consistently augmented with time (Fig. .6B,F). LPS-triggered IL-6 release was detected after 12 and 24 h (Fig. .6E). LPS-induced cytokine release was significantly decreased in TLR4^−/−^ astroglia (Fig. .6B,D,F).

## DISCUSSION

We show that TLR4 in primary murine microglia mediates AS-dependent microglial activation, including the phagocytic activity and the release of pro-inflammatory cytokines and ROS. Furthermore, we demonstrate that TLR4 ablation leads to suppression of the pro-inflammatory response upon AS treatment in astroglia; however, the uptake of AS by astroglial cells is not dependent on TLR4 expression. The comparison of three different AS forms (full length soluble, fibrillar, and C-terminally truncated) on TLR4-dependent glial activation indicates certain variability pointing towards the C-terminally truncated form as the most potent inducer of reactive microglial and astroglial phenotypes.

Previous studies described the involvement of microglial cells in the clearance of AS (Lee et al.,^2008^; Liu et al.,^2007^). Recently, we demonstrated that the ablation of TLR4 in mice overexpressing AS led to AS accumulation in the brain, associated with diminished clearance of AS by microglia and further related to motor impairment and neurodegeneration (Stefanova et al.,^2011^). *Invitro* analysis confirmed TLR4-dependent uptake of AS by microglia (Stefanova et al.,^2011^). In this study, we expand these findings showing for the first time that TLR4 plays a fundamental role in AS-dependent activation of microglial cells. Various studies have demonstrated that ASP are characterized by microgliosis (Gerhard et al.,^2003^,^2006^) and that AS leads to microglial activation (Klegeris et al.,^2008^; Roodveldt et al.,^2010^; Su et al.,^2008^). In accordance with these data, we show that AS is an important stimulus of microglial activation. Furthermore, we demonstrate that the different AS forms lead to different activation profiles of microglia (Table 1). The C-terminally truncated AS form appears to be the strongest activator of microglia as compared with full length soluble or fibrillized AS. Treatment of microglia with tAS leads to a significant increase of ROS production and release of IL-6, CXCL1, and TNF-α. Our results suggest that tAS which was reported previously as a component of Lewy bodies and glial cytoplasmic inclusions (Baba et al.,^1998^; Gai et al.,^1999^; Prasad et al.,^2012^) may play an important pathogenic role to accelerate neuroinflammation and oxidative stress in ASP. Microglia treated with sAS and fAS also present an augmented ROS production and phagocytic activity. In contrast to the findings of Park and colleagues (Park et al.,^2008^), who suggest that fAS inhibited phagocytosis by microglia, we did not detect differences regarding the effects of sAS or fAS on the phagocytic activity of primary murine microglia. This difference may be due to the different type of microglia model used in the experiments (Park et al.,^2008^). Various studies reported microglial cytokine release upon AS treatment (Alvarez-Erviti et al.,^2011^; Klegeris et al.,^2008^; Rojanathammanee et al.,^2011^; Roodveldt et al.,^2010^; Stefanova et al.,^2011^), and elevated cytokine levels in human post-mortem brains of ASP (Imamura et al.,^2005^). We propose that cytokine release by primary microglia is dependent on the AS form and presents with a different temporal profile. Microglia seem to react immediately with TNF-α release after sensing sAS or tAS in the medium, however sAS did not create a long-lasting TNF-α response in comparison to tAS. The treatment with fAS induced only transient TNF-α release detectable 12-h after treatment. Moreover, tAS was the only AS form leading to IL-6 release after 24 h and to continuous CXCL1 release detectable already after 2-h exposure and increasing over time. Cytokine release was associated with NF-κB nuclear translocation in microglia treated with AS. TLR4 deficient microglia did not feature NF-κB translocation from the cytoplasm to the nucleus, corresponding to a significant decrease of cytokine release triggered by AS exposure, respectively. However, not only cytokine release by TLR4 deficient microglia was decreased, but also ROS production and phagocytic activity were significantly reduced upon AS treatment. Therefore, we demonstrate now that TLR4 is a major receptor mediating the immediate innate immune response and also oxidative stress of microglial cells upon AS-treatment, similar to the role of TLR4 on the activation of microglia upon Aβ treatment (Reed-Geaghan et al.,^2009^).

**Table 1:** Activation profile of TLR4^+/+^ and TLR4^−/−^ microglia and astroglia

	TLR4+/+				TLR4^−/−^					
MG	AG	MG	AG
Untreated vs. sAS					
ROS release		↑↑	↑↑	−	−
TNF−α release	2 h	↑↑↑	−	−	−
12 h	↑↑↑	↑↑↑	−	−
24 h	−	−	−	−
IL-6 release	2 h	−	−	−	−
12 h	−	−	−	−
24 h	−	−	−	−
CXCL1 release	2 h	−	−	−	−
12 h	−	−	−	−
24 h	−	−	−	−
Untreated vs fAS			
ROS release		↑↑↑	↑↑↑	−	−
TNF-α release	2 h	−	−	−	−
12 h	↑	−	−	−
24 h	−	−	−	−
IL-6 release	2 h	−	−	−	−
12 h	−	−	−	−
24 h	−	−	−	−
CXCL1 release	2 h	−	−	−	−
12 h	−	−	−	−
24 h	−	−	−	−
Untreated vs tAS			
ROS release		↑↑↑	↑	−	−
TNF-α release	2 h	↑↑↑	−	−	−
12 h	↑↑↑	↑↑↑	−	−
24 h	↑↑↑	↑↑↑	−	−
IL-6 release	2 h	−	−	−	−
12 h	−	↑↑	−	−
24 h	↑↑↑	↑↑↑	−	−
CXCL1 release	2 h	↑↑↑	−	−	−
12 h	↑↑↑	↑↑↑	−	−
24 h	↑↑↑	↑↑↑	−	−

Summary of the activation profile of TLR4^+/+^ and TLR4^−/−^ primary murine microglia (MG) and aged TLR4^+/+^ and TLR4^−/−^ primary murine astroglial (AG) cells upon treatment with different AS forms in comparison with untreated microglia and astroglia. ↑↑↑*P* < 0.001, ↑↑*P* < 0.01, ↑ *P* < 0.05, -*P* > 0.05. sAS, full length soluble α-synuclein; fAS, full length fibrillar α-synuclein; tAS, C-terminally truncated α-synuclein.

We further analyzed the role of TLR4 and AS on the reactivity of astroglial cells. Supporting previous findings of endocytosis-dependent uptake of cell-derived extracellular AS by astroglia (Lee et al.,2010c), we report now the uptake of different forms of recombinant AS (sAS, fAS and tAS) by astroglia. Two hours after AS incubation, astroglial cells showed incorporation of AS in the cytoplasm, independent of TLR4 expression, suggesting that different operative mechanisms control the uptake of AS by microglia and astroglia. Our observations on the inflammatory profile of AS-activated astroglia confirmed previous studies that demonstrated IL-6 release by human astroglia after AS treatment (Klegeris et al.,^2006^), and TNF-α and CXCL1 release by astroglia exposed to neuronal AS (Lee et al.,2010c). We show that incubation with tAS caused an increased and significant release of pro-inflammatory cytokines after 12 h, including TNF-α, IL-6 and the chemokine CXCL1. Alternatively, exposure of astroglia to sAS resulted in a significant increase of TNF-α production after 12 h. However, in our experimental system fAS did not trigger pro-inflammatory response in aged astroglia, but induced significant production of ROS similar to sAS and tAS. The observed augmented ROS production suggests the possibility of astroglia-mediated oxidative stress in ASP. In various studies, TLR4 expression on astroglia was previously demonstrated (Alfonso-Loeches et al.,^2010^; Bowman et al.,^2003^; Bsibsi et al.,^2002^; Carpentier et al.,^2005^; El-Hage et al.,^2011^; Gorina et al.,^2011^). We verified TLR4 expression in the astroglial cell cultures by immunocytochemistry. Moreover, we now demonstrate that TLR4^+/+^ compared with TLR4 ablated astroglia present a differing activation profile (Table 1). TLR4 deficient astroglia showed a significant decrease of ROS production. Similar to decreased ROS levels, cytokine release upon AS treatment by TLR4^−/−^ astroglia was diminished. However, at 24 h of treatment a tendency of higher TNF-α, IL-6 and CXCL1 production by TLR4^−/−^ astroglia as compared with untreated astroglia was detected, suggesting that a prolonged AS exposure may lead to a delayed cytokine/chemokine release by TLR4 deficient astroglia. In support of this hypothesis, we recently demonstrated that MSA transgenic mice with TLR4 knock-down displayed an enhanced accumulation of AS in midbrain and forebrain associated with increased levels of TNF-α (Stefanova et al.,^2011^). Taken together, these observations suggest that the absence of TLR4 on astroglial cells may postpone the AS-dependent activation of astroglia. However, long-term studies using primary cells in cell culture are difficult to accomplish, therefore further in vivo studies are necessary to understand the exact mechanisms of AS-dependent astroglial activation.

In conclusion, in this study we demonstrate that TLR4 is essential for the AS-dependent activation of microglial cells, including phagocytic activity, release of pro-inflammatory cytokines and ROS. The role of TLR4 on astroglial cells seems to be more complex. Unlike microglia, astroglial AS uptake is not dependent on TLR4. However, the activation profile of astroglia proposed in this study suggests an involvement of TLR4 insofar as TLR4 deficiency may suppress the activation of astroglia upon AS treatment. TLR4 as a target to modify the progression of neurodegenerative diseases proves to be a complex issue. By TLR4 suppression microglial activation may be reduced, but this also leads to impaired phagocytosis of neuronal debris and extracellular AS. Furthermore, the predicted impairment of AS phagocytosis by microglia may increase the amount of extracellular AS which may get incorporated by astroglia leading to oxidative stress, delayed inflammatory reaction and possibly enhanced AS inclusion pathology in ASP. Indeed, several other TLRs may interfere with the observed microglial and astroglial responses to AS (Carpentier et al.,^2005^) and will need to be addressed in future studies. This study provides specifically new insights into the mechanisms of TLR4 on glial activation in ASP, expanding the knowledge towards possible new disease modifying targets.

## Notes

Supporting Information Figure 1 Murine primary TL4+/+ astroglia were fixed with 4% paraformaldehyde, immunostained for GFAP (green) and counterstained with TLR4 (red). Co-localization of GFAP and TLR4 confirmed TLR4 expression on TLR4+/+ astroglia (scale bar = 20 μm).
