# Supplementary figures and images for: Toll-like receptor 4 is required for α-synuclein dependent activation of microglia and astroglia
Source: Glia. 2013 Mar;61(3):349–60. doi: 10.1002/glia.22437 (PMC3568908; doi:10.1002/glia.22437)

GFAP

TLR4

Merge

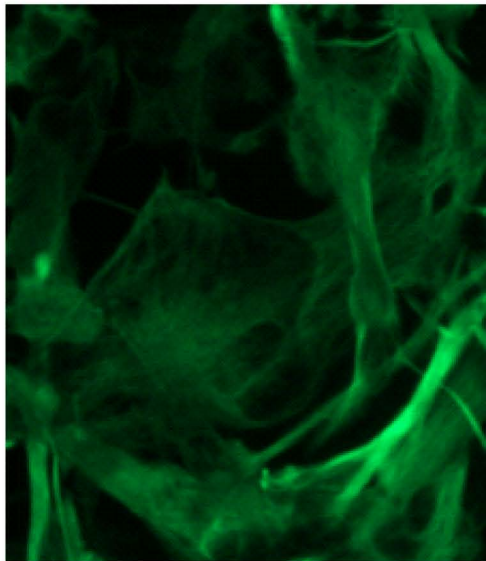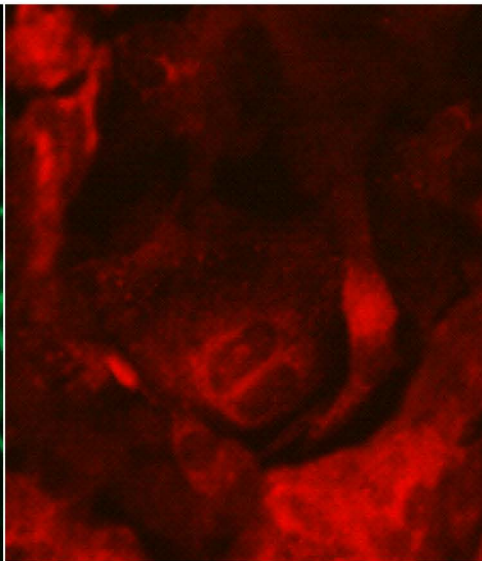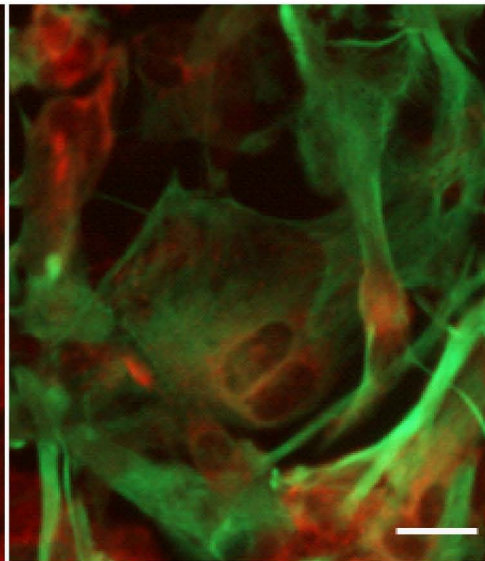

Supplement: Supporting Information Figure 1 — Murine primary TL4+/+ astroglia were fixed with 4% paraformaldehyde, immunostained for GFAP (green) and counterstained with TLR4 (red). Co-localization of GFAP and TLR4 confirmed TLR4 expression on TLR4+/+ astroglia (scale bar = 20 μm). [file glia0061-0349-sd1.pdf]
